# Construction of a Two-Gene Immunogenomic-Related Prognostic Signature in Lung Squamous Cell Carcinoma

**DOI:** 10.3389/fmolb.2022.867494

**Published:** 2022-04-08

**Authors:** Xiaoting Zhang, Jing Xiao, Xian Fu, Guicheng Qin, Mengli Yu, Guihong Chen, Xiaofeng Li

**Affiliations:** ^1^ Shenzhen Bao’an District Songgang People’s Hospital, Shenzhen, China; ^2^ School of Pharmaceutical Sciences, Guangzhou Medical University, Guangzhou, China; ^3^ Department of Laboratory Medicine, Peking University Shenzhen Hospital, Shenzhen, China

**Keywords:** lung squamous cell carcinoma, immune-related genes, TCGA, prognosis risk model, FGA, CSF2

## Abstract

Lung cancer has the highest tumor incidence in China. Lung squamous cell carcinoma (LUSC) is the most common type, accounting for 40–51% of primary lung cancers. LUSC is slow in growth and late in metastasis. Immune-related genes (IRGs) and immune infiltrating cells play a vital role in the clinical outcomes of LUSC. It is important to systematically study its immune gene map to help the prognosis of cancer patients. In this study, we combined the prognostic landscape and expression status of IRGs downloaded from the TCGA and InnatedDB databases and systematically analyzed the prognostic information of LUSC patients to obtain IRGs. After systematically exploring the survival analysis, prognosis-related genes were found, and the PPI network revealed that a total of 11 genes were hub genes. A two-gene prognosis risk model was established by multivariate Cox analysis. Two IRGs were closely correlated with the prognosis of LUSC. Based on these two genes, a new independent prognostic risk model was established, and this model was further verified in the GEO database. Moreover, the risk score of the model was correlated with sex, survival status, and lymphatic metastasis in LUSC patients, and the predictive risk of the prognostic risk model was significantly positively correlated with five kinds of immune cells (CD4 T cells, CD8 T cells, neutrophils, macrophages, and dendritic cells). This study comprehensively analyzed immunogenomics and presented immune-related prognostic biomarkers for LUSC.

## Introduction

Lung cancer is one of the most common malignancies worldwide and is caused by malignant cancers (Sung et al., 2021). Lung cancer can be divided into two typical subtypes, small cell lung cancer (SCLC) and non-small-cell lung cancer (NSCLC), and NSCLC, which accounts for approximately 83% of lung cancer patients, is further categorized into lung squamous cell carcinoma (LUSC) and lung adenocarcinoma (LUAD) according to histological classification and pathogenesis (van Meerbeeck et al., 2011). Moreover, LUSC accounts for approximately 30% of NSCLC cases and has an unsatisfactory prognosis due to the lack of effective targeted treatment. NSCLC patients are in the advanced stage once diagnosed, and their 5-year survival rate is remarkably lower than that of early-stage patients (Tarver 2012; Fujimoto and Wistuba 2014; Wang BY. et al., 2020).

Currently, many treatment modalities, such as surgical resection, radiotherapy, chemotherapy, targeted therapy, and immunotherapy, have been applied to treat lung cancer (Lemjabbar-Alaoui et al., 2015). Surgical resection depends on the advanced stage of cancer. For early-stage NSCLC patients, surgical resection is often the first choice, while for advanced-stage patients whose tumors cannot be removed by surgery, a targeted and more effective therapy depending on molecular tumor characteristics or immunotherapy combined with chemotherapy is more useful (Zarogoulidis et al., 2013). Lung cancer is characterized as a highly complex and heterogeneous disease and several causes are related to lung cancer mortality, such as environmental factors and tobacco smoking habits, which are two major factors in lung cancer (Malhotra et al., 2016; Lipfert and Wyzga 2019). Currently, an increasing number of studies have indicated that some molecular characteristics and signaling pathway targets also contribute to lung cancer development. Abnormal genetic alterations, such as epidermal growth factor receptor (EGFR) (Castellanos et al., 2016; Rodriguezcanales et al., 2016; Liu et al., 2017) and epidermal growth factor receptor 2 (HER2) (Pillai et al., 2014; Catherine et al., 2017), are frequently described to participate in the development of lung cancer.

Thus, to provide a better prognosis suggestion and prediction for patients receiving more precise treatment for LUSC, a predictive prognosis model based on prognostic gene biomarkers is urgently needed. Several clinical information and gene expression datasets can be found in public databases, such as Gene Expression Omnibus (GEO) and The Cancer Genome Atlas (TCGA), providing possibilities for bioinformatics analysis in the identification of novel promising biomarkers for cancer treatment. For example, Gao et al. (2020) identified a five-gene-based risk model signature (CCNA2, AURKA, AURKB, and FEN1) to predict the prognosis status of LUSC patients. In addition, the prognostic value of potential immune-related genes (IRGs) was explored to utilize personalized immune signals for optimal prognostic evaluations in nonsquamous NSCLC patients. Although it was reported recently that an 11-gene-related prognostic model, including CXCL5, MMP12, PLAU, ELN, JUN, RNASE7, JAG1, SPP1, AGTR2, FGFR4, and TNFRSF18, performed well in the prognostic forecast in LUSC (Zhang, et al., 2020a), the model was still too complex and inconvenient for clinical diagnosis and practical application. Thus, a simpler and more feasible prognostic risk model that can reveal the prognostic significance and clinical correlation of IRGs in LUSC needs to be further explored.

Accordingly, in this paper, we focused on the prognostic analysis of LUSC patients. We systematically analyzed the clinical information and survival status of LUSC patients downloaded from the TCGA, InnatedDB, and GEO databases to establish a two-gene prognosis risk model for LUSC patients, which also has an excellent ability to predict immune cell infiltration. Our research aimed to provide the potential to provide better treatment advice for LUSC patients.

## Materials and Methods

### Data Collection

**FIGURE 1:**
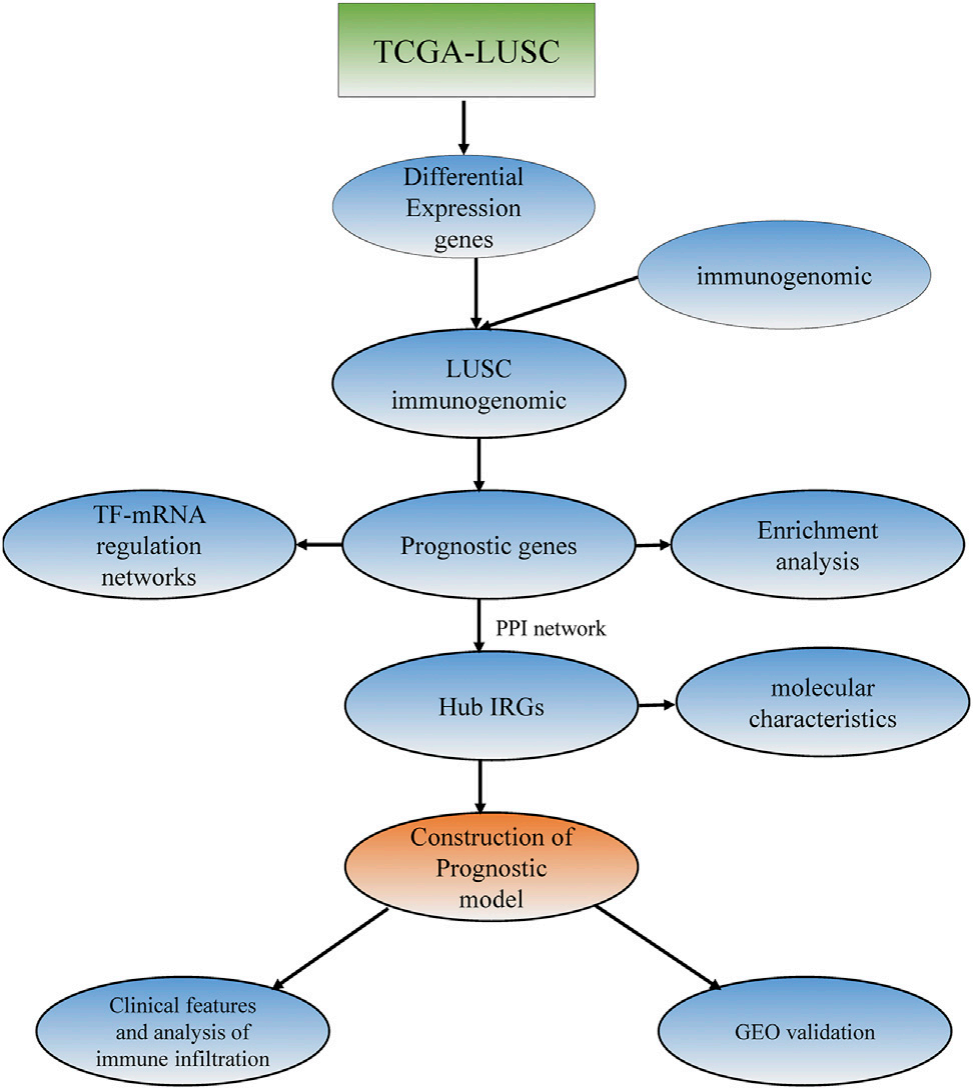
Workflow of the bioinformatics analysis.

The gene expression dataset and corresponding clinical information for LUSC containing 489 LUSC tissues and 49 normal tissues were downloaded from the TCGA database (https://tcga-data.nci.nih.gov/tcga/) (Table 1) (Tomczak et al., 2015). The dataset (accession number: GSE74777) with gene expression information and survival information data, containing a total of 109 tumor samples, was downloaded from the Gene Expression Omnibus (GEO) (https://www.ncbi.nlm.nih.gov/geo/), which was used as a verification set. A total of 1,697 genes from the InnateDB database (https://www.innatedb.ca) (Bhattacharya et al., 2014) and 1811 genes from the Immport database (https://www.immport.org) were identified as IRGs. The research procedure is shown in Figure 1.

**TABLE 1:** Clinical characteristics of TCGA sample.

Parameter	Subtype	Patients
Age	<70	269
	≥70	215
	Unknown	5
Gender	Women	127
	Men	362
M stage	M0	402
	M1	7
	MX	76
	Unknown	4
Cancer status	Tumor free	249
	With tumor	152
	Unknown	88
T stage	T1	49
	T1a	24
	T1b	38
	T2	168
	T2a	86
	T2b	32
	T3	69
	T4	23
N stage	N0	313
	N1	126
	N2	40
	N3	5
	NX	5

### Analysis of IRGs in LUSC

To obtain the differentially expressed genes (DEGs), 489 LUSC tissues and 49 normal tissues shared by the TCGA database were analyzed by the R software limma package, taking a log2 | fold change|>1 and a false discovery rate (FDR) < 0.01 as screening criteria. The similarity genes in DEGs and 3,187 IRGs searched from the InnateDB and Immport databases were further compared to obtain a series of IRGs in LUSC.

### Survival Analysis

Multivariate survival analysis in 489 patients with LUSC was performed to investigate the relationship between IRGs and prognosis using the R software survival package. Then, the prognostic risk model of LUSC was built on the basis of the multivariate coefficiency multiplied by expression data. The Kaplan-Meier method and long-rank test, taking the long-rank test and *p* value < 0.05 as statistically significant, were used to determine the relationship between overall survival (OS) and survival-related prognostic genes. The property of prognostic factors was computed by the survival ROC by computing the area under the curve (AUC) (Heagerty et al., 2000). Moreover, to investigate the degree of precision of the prognostic risk model in predicting the survival status of LUSC patients, calibration curves and ROC curves were carried out.

### Functional and Pathway Enrichment Analysis

To further verify whether the IRGs participated in functional regulation and pathways, GO and KEGG enrichment analyses were performed using the R programming language. First, up- and downregulated genes were both analyzed, and a *p* value < 0.05 was considered the threshold value. Furthermore, a series of gene functional enrichment analyses, including GO and KEGG analyses, were conducted to identify the major biological attributes. Finally, the GO-plot package was used to generate a bar chart of GO and a circle diagram of KEGG.

### Transcription Factor-mRNA Interaction Analysis

The regulatory relationship between transcription factors and mRNAs was downloaded from the TRRUST v2 database (www.grnpedia.org/trrust) (Mei et al., 2017). Those transcription factors that interacted with hub genes were selected to construct the interaction network to establish a correlation between IRGs and TFs, which was further exhibited by Cytoscape.

### Recognization of Hub Genes and Verification of Their Molecular Characteristics

The IRGs were analyzed with the STRING database (https://string-db.org/) to establish a protein–protein network and further reorganized by Cytoscape software. Related hub genes were found according to the node degree in the network, and the mutation information of those hub genes was obtained based on the Cbioportal website (http:/www.cbioportal.org/) (Cerami et al., 2012; Gao et al., 2013).

### Establishment and Verification of the Prognostic Risk Model

A multivariate Cox proportional hazards regression analysis was performed using the candidate prognostic genes to build a risk prognostic risk model. Furthermore, the median value of 107 cancer samples from the accession set GSE74777 in the GEO database, which was utilized as the training dataset, was calculated separately to evaluate the prediction accuracy of the prognostic risk model. *p* < 0.05 was considered significant.

### The Relationship Between the Prognostic Predictive Model and Other Clinical Characteristics

The infiltration status of six kinds of immune-related cells from cancer tissues was calculated on the TIMER website (https://cistrome.shinyaoos.io/timer/). T tests and Kruskal–Wallis tests were performed to identify the relationship between the prognostic predictive model and other infiltration clinical factors separately. *p* < 0.05 indicates statistical significance.

## Results

### Identification of Differentially Expressed Immune-Related Genes

The number of IRGs was found to be 3,187 by taking the union of genes downloaded from the InnateDB and Immport datasets, which were 1,697 and 1811, respectively.

**FIGURE 2:**
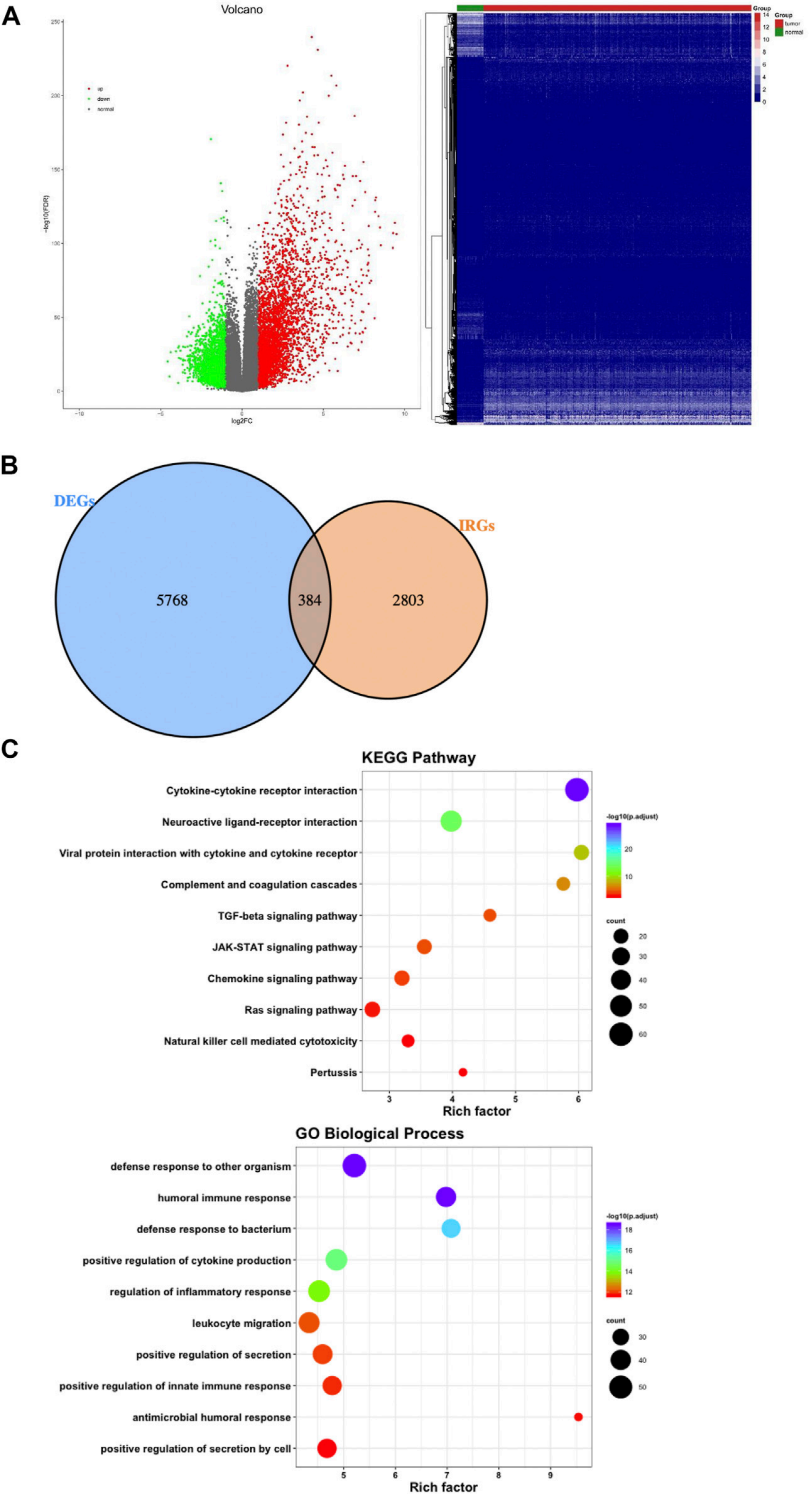
Differentially expressed genes and functional enrichment analysis of differentially expressed IRGs. **(A)** Volcano plot and heatmap demonstrating differentially expressed genes between lung squamous cell carcinoma (LUSC) and nontumor tissues. Green and red dots represent differentially expressed genes, and gray dots represent genes that were not differentially expressed. **(B)** Venn diagram shows the common differentially expressed genes and immune-related genes. **(C)** Significantly enriched KEGG pathways and enriched GO terms based on biological processes. GO = gene ontology, IRGs = immune-related genes, KEGG = Kyoto Encyclopedia of Genes and Genomes.

There were 1,697 IRGs downloaded from the InnateDB database (https://www.innatedb.ca/) and 1811 IRGs downloaded from the Immport database (https://www.immport.org). We combined the two results and found a total of 3,187 IRGs. A total of 6,152 DEGs were screened after screening the gene expression data of 489 LUSC tissues and 49 normal tissues by taking log2 | fold change|>1 and false discovery rate (FDR) < 0.01. When comparing the gene expression levels in the normal tissues, 4,102 genes were upregulated, and 2050 genes were downregulated (Figure 2A).

Combining 6,152 differentially expressed genes with 3,187 IRGs, the 384 genes in the intersection were considered LUSC IRGs (Figure 2B). Functional and pathway enrichment analysis of the 384 IRGs revealed their role in a biological network related to innate immune regulation and humoral immune reaction regulation (Figure 2C).

### Identification of Prognosis-Related Genes

**FIGURE 3:**
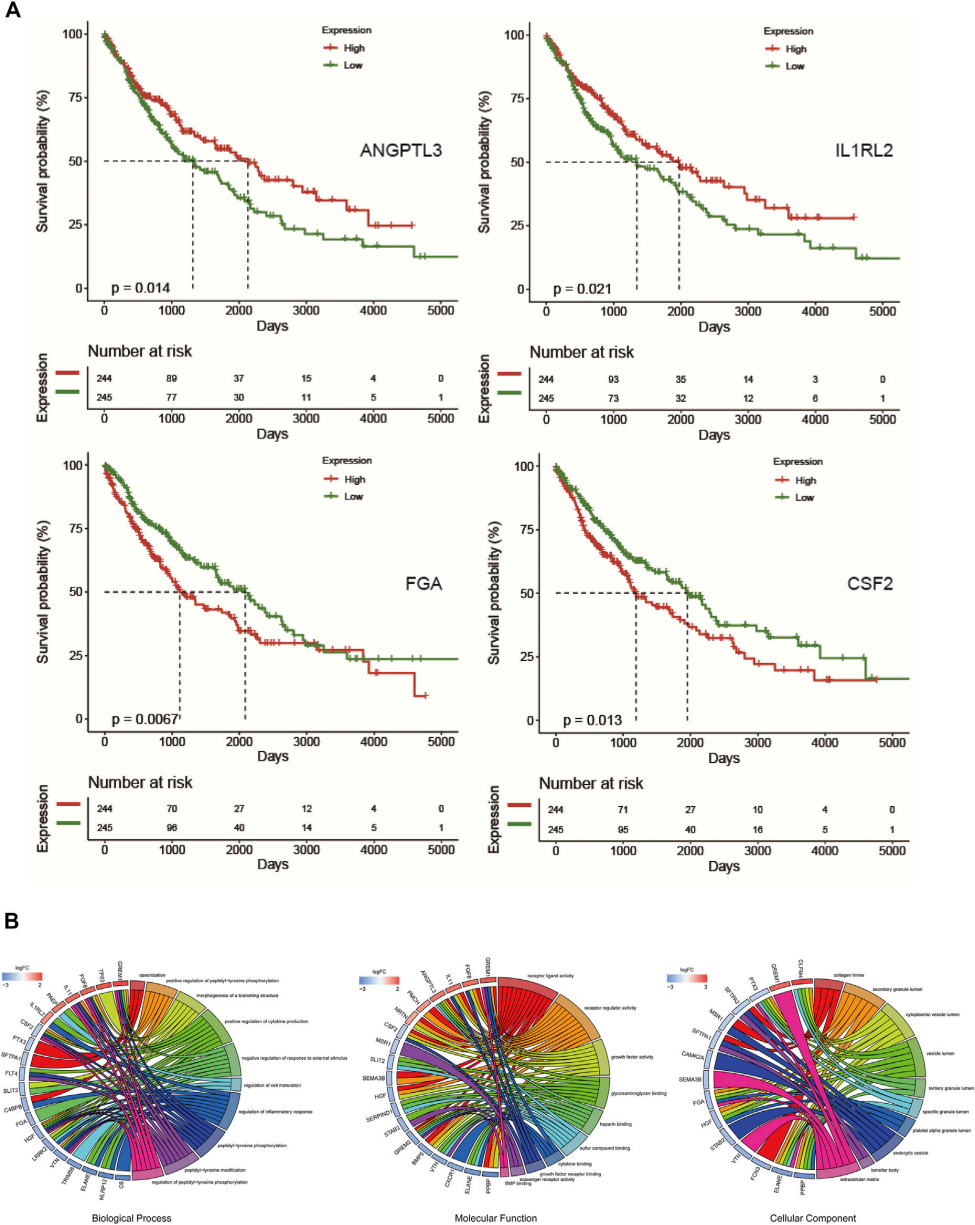
Overall survival-related genes and enrichment analysis. **(A)** Overall survival curve of ANGPTL3, IL1RL2, FGA, and CSF2. **(B)** GO enrichment analysis of 46 overall survival-associated genes.

To select genes related to the prognosis of LUSC, 384 IRGs were divided into high-expression and low-expression groups according to the median expression level (Supplementary Table S1). After performing a survival analysis, 46 survival-related prognostic genes were found (Supplementary Table S2). Some of the gene survival curves are shown in Figure 3A. Furthermore, functional and pathway enrichment analyses were carried out and revealed that these genes were involved in cell factor regulation and cell maturation regulation (Figure 3B).

### Construction of the PPI Network and Identification of Hub IRGs

**FIGURE 4:**
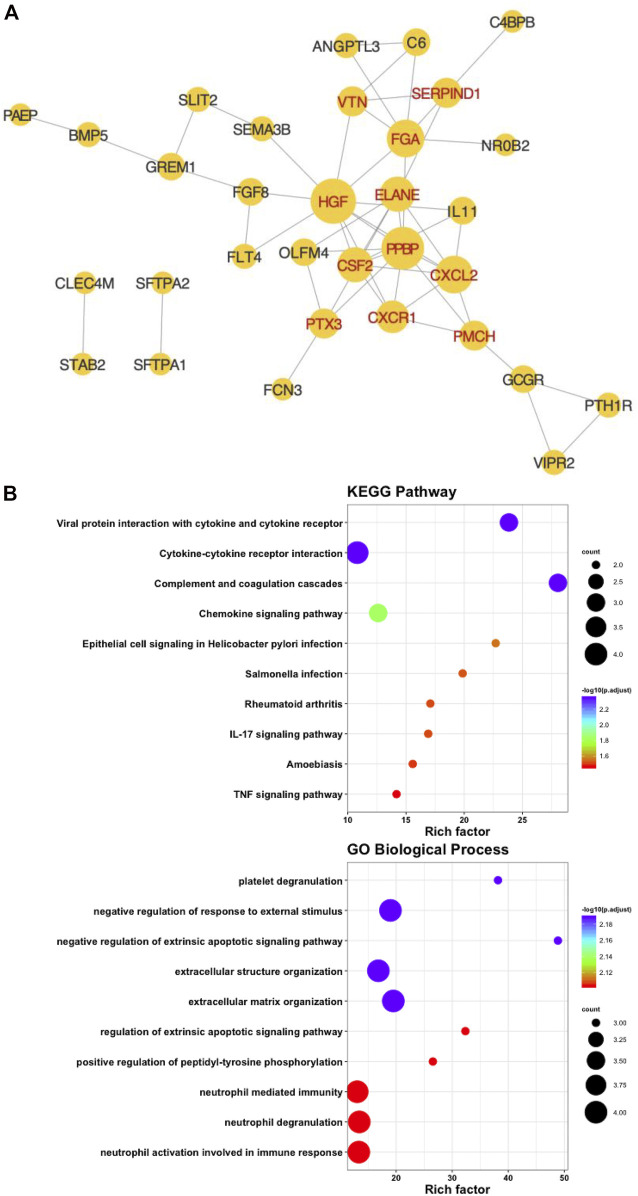
Identification of hub immune-associated genes. **(A)** Protein-protein interaction network of hub IRGs. **(B)** Kyoto Encyclopedia of Genes and GO Biological Process analysis of hub IRGs.

We placed forty-six prognostic IRGs into the STRING database to investigate their value in the protein–protein interaction network, and we reconstructed them with Cytoscape software. The PPI network showed that it contained 32° and 53 sides. A total of 11 hub IRGs were identified by calculating the degree of every prognostic IRG and taking degree>3 (Figure 4A). Functional and pathway enrichment analyses were performed and indicated that the hub IRGs were involved in cell factor-receptor interactions and the tumor necrosis signaling pathway (Figure 4B).

**FIGURE 5:**
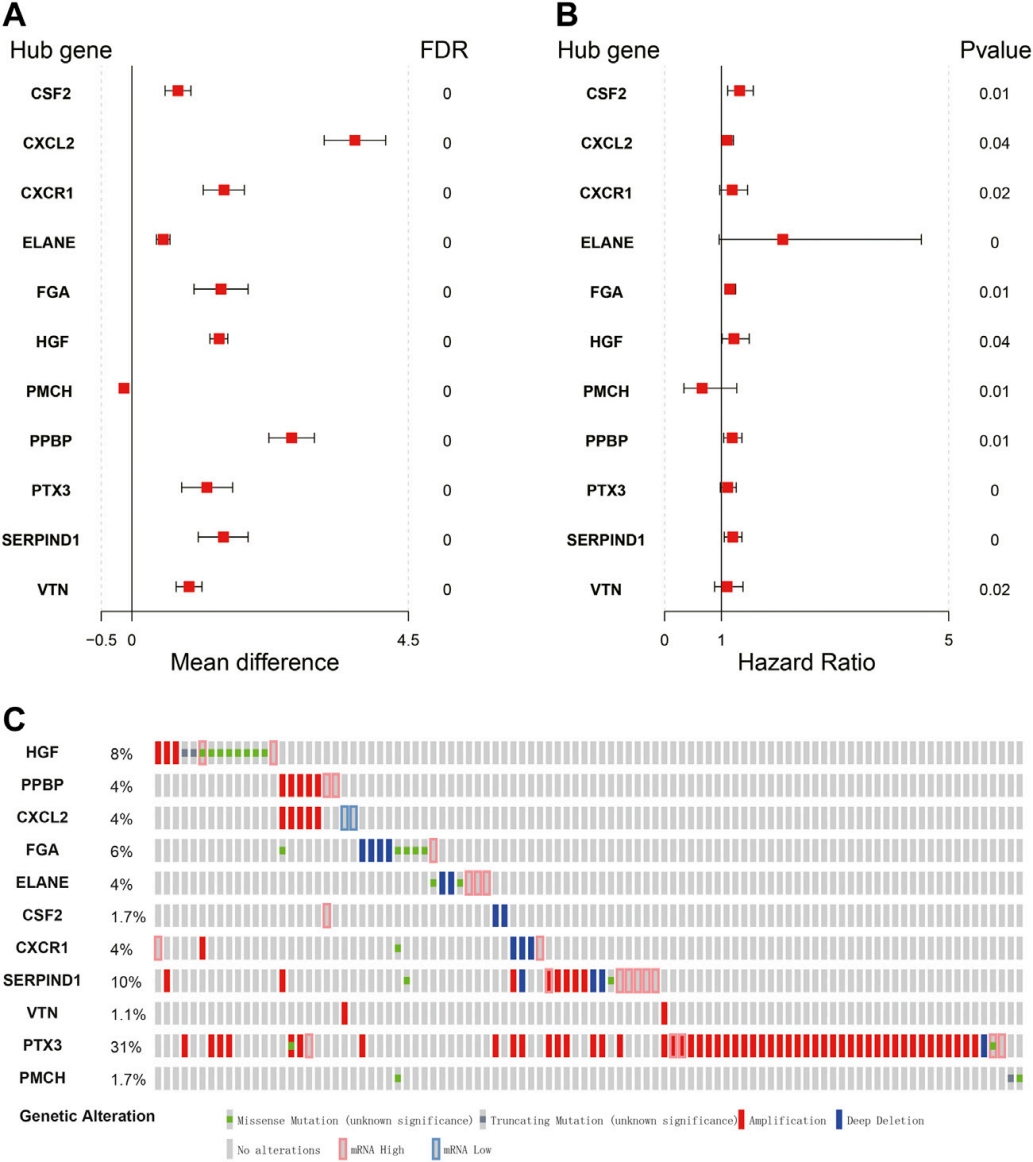
Expression profiles and mutation landscape of hub immune-related genes. **(A,B)** Forest plot of the mean difference showing gene differences between LUSC and nontumor samples (left panel). Forest plot of hazard ratios showing the prognostic values of genes (right panel). **(C)** The diagram reflects the mutation type and frequency of risk genes.

The expression signature of hub IRGs and the HR forest graph showed that most of those hub IRGs were upregulated and were risk factors (Figures 5A,B). Furthermore, we obtained their genetic mutation information from the Cbioportal website to explore their biological molecular characteristics. We found that amplification and mRNA upregulation were the two most common mutation types (Figure 5C).

### Transcription Factor Regulatory Network

**FIGURE 6:**
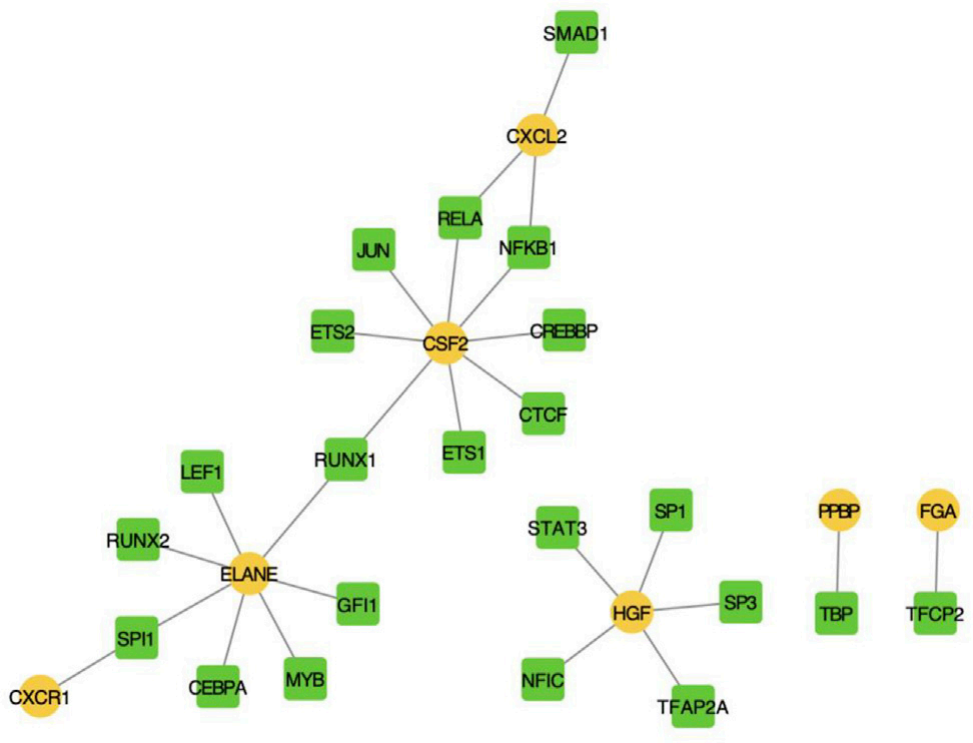
Transcription factor-mediated regulatory network.

We screened human TF-mRNA regulation from the TRRUST v2 database and showed that many transcription factors interacted with hub IRGs, including MYB, SP1, and SP3 (Figure 6).

### Construction of a Prognostic Risk Based on Hub IRGs

A multivariate Cox analysis was performed to explore the associations between the hub IRGs and the predicative signature, and two key genes (FGA and CSF2) were finally used as independent prognostic factors in establishing the prognostic risk model of LUSC patients. The prognostic risk model of LUSC based on two independent genes was as follows: Risk Score = EXPFGA*0.1340112 + EXPCSF2*0.2431801.

**FIGURE 7:**
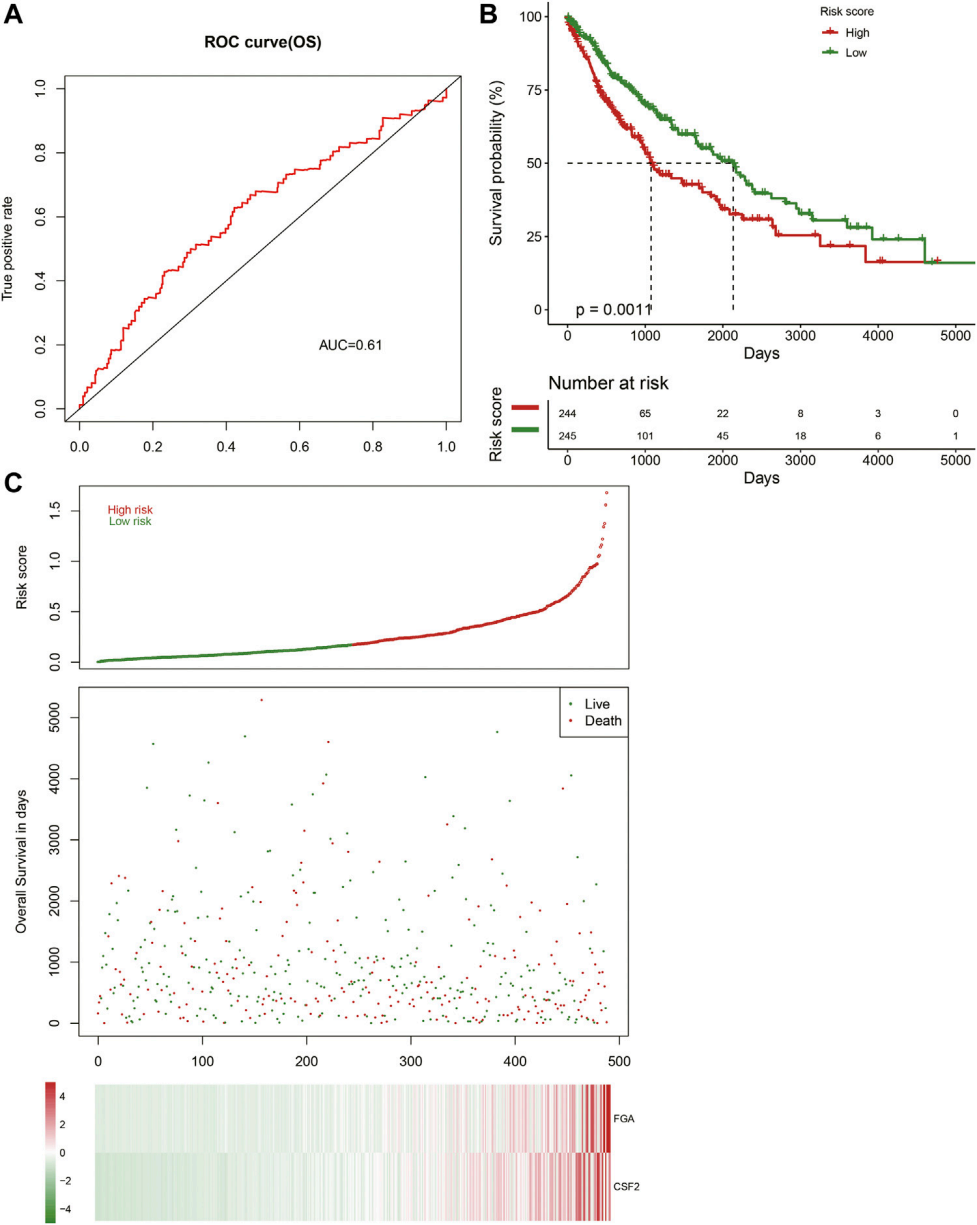
Construction of the prognostic model. **(A)** Receiver operating characteristic (ROC) curve showing the prognostic value of the risk signature. **(B)** Patients in the high-risk group demonstrate shorter overall survival. **(C)** Survival conditions of LUSC patients.

The AUC value of the prognostic risk model was 0.61 when predicting LUSC patient samples at 1 year, indicating that the expression of FGA and CSF2 could predict the prognosis status of LUSC patients (Figure 7A). We calculated the prognostic risk value of individual patients and classified them into high-risk and low-risk groups on the basis of their expected risk value in the prognostic risk model, which showed that there was a remarkable difference between the two groups (Figures 7B,C).

### Verification of the Prognostic Risk Model

**FIGURE 8:**
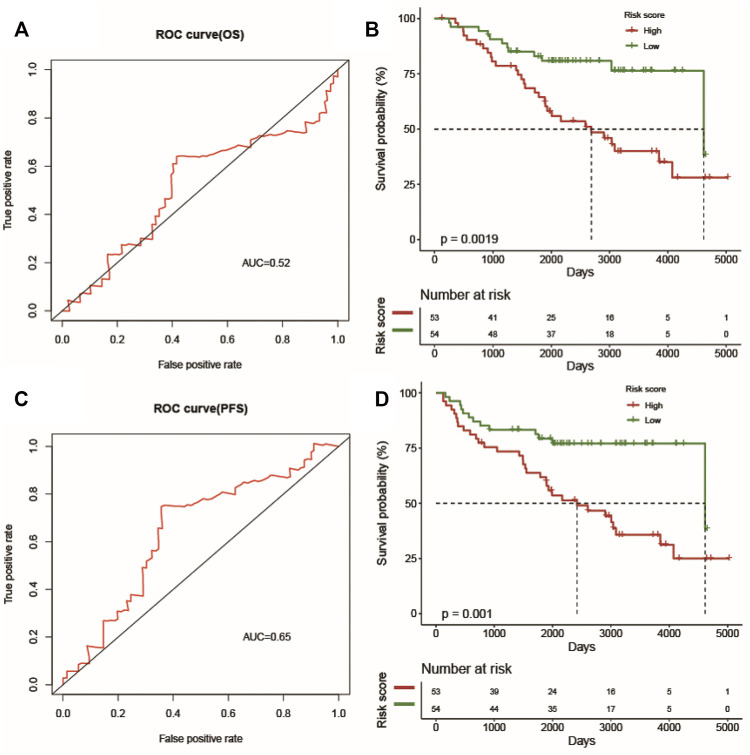
The prognostic value of the prognostic index. The ROC curve verifies the accuracy of the combined model in predicting the 1-year survival rates **(A)** and progression-free survival **(C)** of LUSC patients. Patients in the high-risk group suffered shorter survival rates **(B)** and progression-free survival **(D)**.

To further validate the predicative ability of the prognostic risk model of LUSC, the GSE74777 dataset, including 107 cancer samples from the GEO database, was collected. The AUC (OS) and AUC (PFS) values of each sample were calculated, and the predicative values of the prognostic risk model at 1 year were 0.52 and 0.65, respectively (Figures 8A, C). In addition, we calculated the risk value of every patient and categorized them into high-risk and low-risk groups based on their risk value in the prognostic risk model, which showed that it was significant in both groups (Figures 8B,D) (*p* < 0.05).

### Clinical Utility of the Prognostic Signature

**FIGURE 9:**
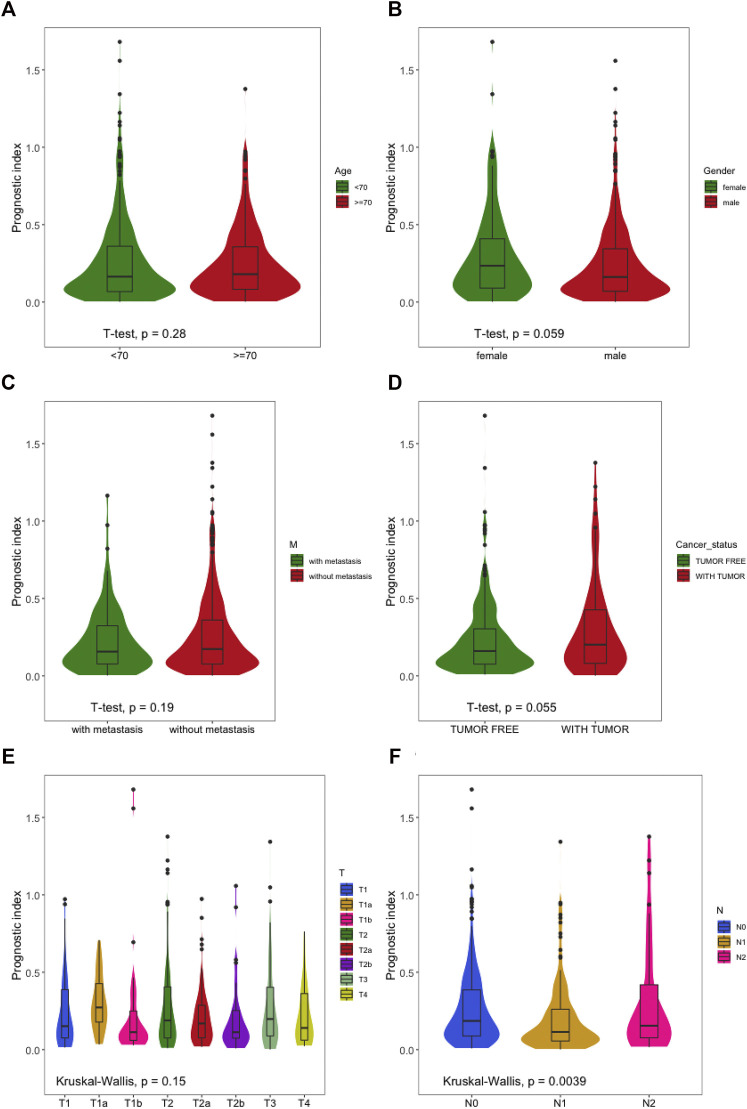
The relationships between the immune-based prognostic index and clinical features. **(A)** Prognostic index and age; **(B)** prognostic index and sex; **(C)** prognostic index and distant metastasis; **(D)** prognostic index and cancer status; **(E)** prognostic index and T stage; **(F)** prognostic index and node metastasis.

We applied T test and Kruskal–Wallis analyses to evaluate the relationship between the prognostic risk model of LUSC and clinical characteristics (age, sex, clinical stage, and metastasis). The statistical tests showed that the patients with LUSC, age, distant metastasis stage T, and M had no significant correlation with the prognostic prediction, while the recurrence risk in different T stages had a relationship with extent (*p* = 0.059/*p* = 0.055) (Figures 9A–E). However, the prognostic risk was significantly associated with lymph node metastasis N (Figure 9F) (*p* < 0.05).

### Immunocyte Infiltration in the LUSC Microenvironment

**FIGURE 10:**
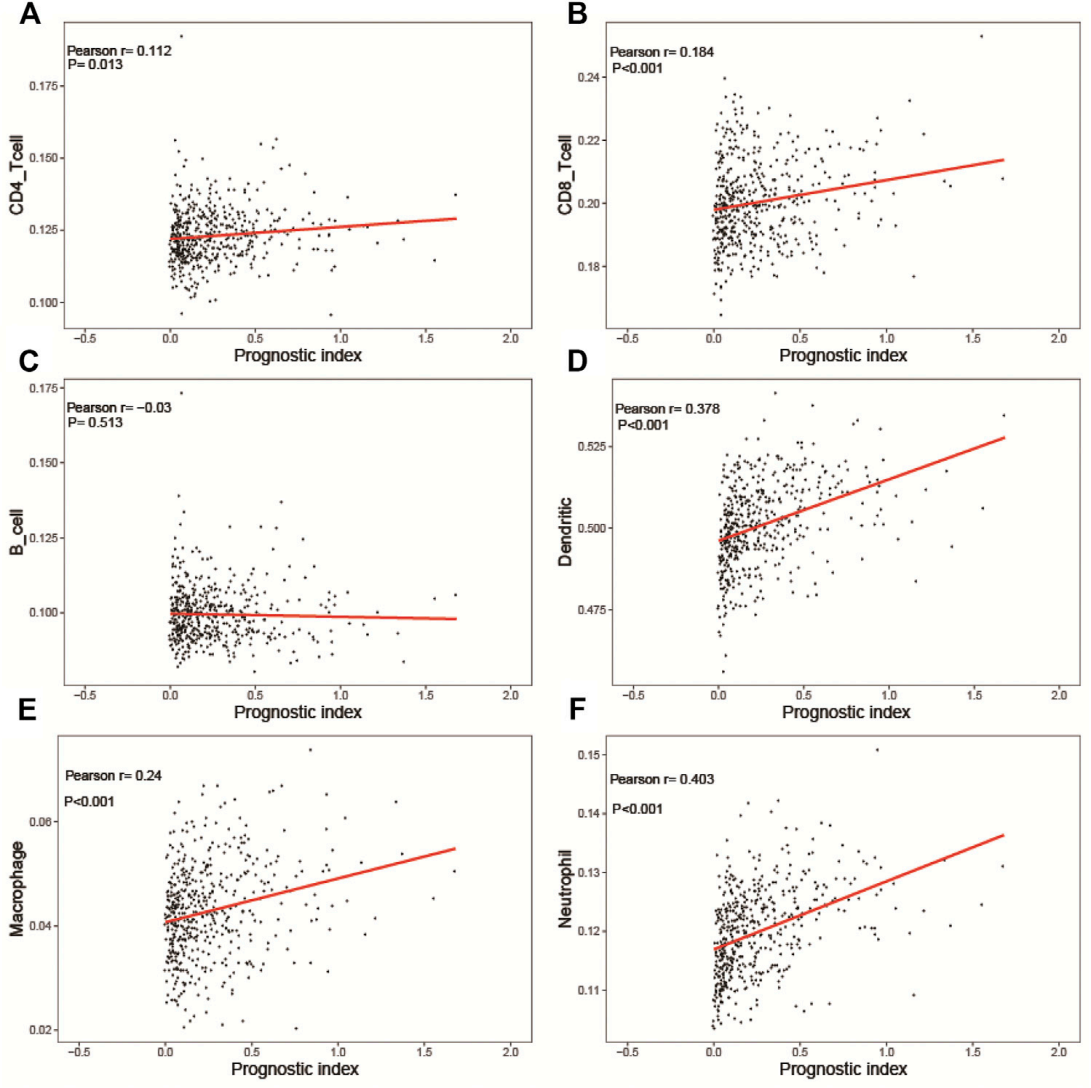
Relationships between the immune-related prognostic index and immune cell infiltration. **(A)** CD4 T cells; **(B)** CD8 T cells; **(C)** B cells; **(D)** dendritic cells; **(E)** macrophages; **(F)** neutrophils.

We calculated the infiltration signature of six kinds of immune-related cells on the Timer website and evaluated the correlation between the predictive risk of the prognostic risk model of LUSC and the infiltration of those cells. The predictive risk of the prognostic risk model was significantly positively correlated with five kinds of immune cells except for B cells (Figure 10C) (CD4 T cells (Figure 10A), CD8 T cells (Figure 10B), neutrophils (Figure 10D), macrophages (Figure 10E), and dendritic cells (Figure 10F). Interestingly, the infiltration status increased when the predictive risk of the prognostic risk model was high.

## Discussion

Lung cancer is heterogeneous and has no effective treatment options when it progresses to the late stage (Leitao et al., 2018). The genomic mutation patterns in LUSC are complex (de Sousa and Carvalho 2018). Mutations in ALK and EGFR have caused huge changes in the treatment of patients with lung adenocarcinoma (Lynch et al., 2004; Paez et al., 2004; Soda et al., 2007). However, ALK fusions or EGFR mutations do not exist in LUSC (Rekhtman et al., 2012). Thus, a novel LUSC biomarker with high specificity and high sensitivity needs to be identified for clinical diagnosis and prognosis. Recently, the importance of IRGs in many cancer immunotherapies has been accepted and is on the rise (Campbell et al., 2016; Faruki et al., 2017). However, an overall genome-wide analysis of LUSC is still needed to explore the clinical significance and even the molecular mechanism. In this study, we showed the effects of IRGs on LUSC clinical significance and explained the molecular features. These IRGs are potentially valuable for clinical characteristics.

To construct a simple and suitable formula to predict the clinical outcomes of LUSC patients, two IRGs (FGA and CSF2) were selected from 11 hub IRGs to establish the risk signature (risk score = EXPFGA*0.1340112 + EXPCSF2*0.2431801). LUSC patients with high-risk values have a poor prognosis, and this risk signature has the ability to distinguish high-risk LUSC patients. Moreover, this signature may be applied as an independent prognostic factor.

FGA encodes the subunit of the coagulation factor fibrinogen, which is a component of the blood clot (Simpson-Haidaris and Rybarczyk 2001; Staton et al., 2003). Mutation of FGA can result in hereditary systemic amyloidosis (Benson, 2005). It was reported that FGA and related fragments are involved in tumor angiogenesis and metastasis. FGA and its catabolite can regulate the overall antigenicity of solid tumors (Wang M. et al., 2020). Importantly, FGA can interact with HBsAg and induce apoptosis in HepG2 cells (Li et al., 2014). It has been reported that FGA inhibits cell proliferation and migration and induces apoptosis in A549 cells. Wang et al*.* (2020) recently found that knockout of fibrinogen alpha increased tumor growth and metastasis by activating the integrin-AKT signaling pathway in lung cancer. Furthermore, the FGA isoform has been shown to be a predictor of targeted therapy in patients with EGFR-mutated lung adenocarcinoma (Shang et al., 2019). Colony stimulating factor 2 (CSF2) is a main factor that regulates the production, differentiation, and function of granulocytes and macrophages (Hamilton 2008; Hamilton and Achuthan 2013). Many previous studies indicated that CSF2 produced by cancer cells is involved in the autocrine regulation of cell growth in human skin, prostate, bladder, melanoma, gastric colon and non-small-cell lung cancer cells (NSCLC) (Oshika et al., 1998; Mueller et al., 1999; Lammel et al., 2012; Yi-Ying et al., 2016). In human glioma cells, CSF2 can promote cell growth and invasion (Sielska et al., 2020). In addition, the expression of these IRGs can be regulated by the transcription factors MYB, SP1, and SP3, which are related to tumor immunity (Beug et al., 2019; Liang et al., 2021; Yu et al., 2021). These findings imply that the identified IRGs or TFs may be therapeutic targets or novel biomarkers for LUSC.

Increasing evidence implies that cancer immune-infiltrating cells are closely related to clinical outcomes (Zhang X. et al., 2020; Liu et al., 2021). In this report, we also explored the correlation between the risk signature and infiltrating immune cells. Significantly, the prognostic index positively correlated with the infiltration of CD4^+^ T cells, CD8^+^ T cells, dendritic cells, macrophages, and neutrophils. These findings suggest that five immune cell types may be a predictor for immune cell infiltration. Interestingly, it has been reported that the early proliferative CD8^+^ T-cell response to PD-1-targeted therapy is correlated with a favorable prognosis in NSCLC patients. Tumor-associated macrophages are widely present in many tumors. It can increase tumor growth, metastasis, invasion, and drug resistance. Tumor-associated macrophage infiltration is closely associated with tumor cell proliferation and survival in patients with LUSC.

Overall, our findings reported that the novel signature (risk score = EXPFGA*0.1340112 + EXPCSF2*0.2431801) could be applied as a prognostic method for LUSC and a potential predictor of immune status in patients with LUSC. Further reliable validation with large-scale sample cohorts is still needed for this signature, which might be valuable for the clinical diagnosis of LUSC in the future.

## Data Availability

The original contributions presented in the study are included in the article/Supplementary Material, further inquiries can be directed to the corresponding authors.
